# A Comparison of Vaccination Policies and Immunity Assessment for Measles Control: Insights from the United States and Japan

**DOI:** 10.3390/v17060861

**Published:** 2025-06-17

**Authors:** Naruhito Otani, Toshiomi Okuno, Toshie Tsuchida, Kaori Ishikawa, Kaoru Ichiki, Takashi Ueda, Satoshi Higasa, Kazuhiko Nakajima

**Affiliations:** 1 Department of Infection Control and Prevention, Hyogo Medical University, Hyogo, Nishinomiya 663-8501, Japan; tsuchida@hyo-med.ac.jp (T.T.); i-kaori@hyo-med.ac.jp (K.I.); ichiki@hyo-med.ac.jp (K.I.); taka76@hyo-med.ac.jp (T.U.); nakajima@hyo-med.ac.jp (K.N.); 2 Department of Public Health, Hyogo Medical University, Hyogo, Nishinomiya 663-8501, Japan; 3 Department of Microbiology, Hyogo Medical University, Hyogo, Nishinomiya 663-8501, Japan; tmokuno@hyo-med.ac.jp; 4 Department of Respiratory Medicine and Hematology, Hyogo Medical University, Hyogo, Nishinomiya 663-8501, Japan; parasol@mua.biglobe.ne.jp

**Keywords:** measles, epidemiology, vaccination policy, immunity assessment, vaccine hesitancy, outbreak control, United States, Japan

## Abstract

Measles is a highly contagious viral disease and remains a global health challenge despite the availability of effective vaccines. Although many regions have successfully eliminated measles, outbreaks continue to occur owing to vaccine hesitancy, inadequate coverage, and imported cases. Differences in epidemiology, vaccination policies, and immunity assessment influence measles control across countries. This paper compares measles epidemiology, vaccination policies, and immunity assessment approaches in the United States and Japan. Data were obtained from surveillance reports, national immunization programs, and peer-reviewed literature. The introduction of the measles vaccine led to substantial reductions in incidence. The United States eliminated measles in 2000 but continues to experience outbreaks due to vaccine hesitancy and imported cases. Japan implemented a two-dose policy in 2006, reducing case numbers; however, sporadic outbreaks among adults persist. In the United States, immunity is primarily assessed using documented vaccination history, whereas in Japan, enzyme immunoassay is commonly used to evaluate immunity status. Despite progress in measles elimination, achieving high vaccination coverage and addressing vaccine hesitancy remain critical challenges. Variations in immunity assessment methods impact surveillance accuracy and outbreak control. Strengthening international collaboration, standardizing assessment protocols, and enhancing public health education are crucial for sustained measles elimination.

## 1. Introduction

Measles is a single-stranded RNA virus classified under the *Morbillivirus* genus within the *Paramyxoviridae* family. It is highly contagious and spreads through droplets, airborne transmission, and direct contact [1]. The measles virus was first successfully isolated in 1954 by American virologist John Enders [2]. Around the same time, Yoshiomi Okuno in Japan independently isolated the virus using a different method, thereby significantly contributing to measles control measures and vaccine development in Japan [3]. Despite significant progress in the prevention of measles, it is still a major public health challenge, with outbreaks occurring even in regions with established immunization programs. The increasing incidence of measles in some high-income countries due to vaccine hesitancy underscores the ongoing challenges in achieving global measles elimination.

Measles is typically diagnosed based on fever, cough, runny nose, and conjunctivitis, followed by the appearance of Koplik spots and a rash. Severe complications include pneumonia, encephalitis, and subacute sclerosing panencephalitis, with infants and immunocompromised individuals at the highest risk of severe disease [4].

Vaccination is still the most effective strategy for the prevention of measles. Since the introduction of the measles vaccine in the United States in 1963, the global incidence of measles has declined significantly [5]. In Japan, the vaccine was incorporated into the routine immunization program in 1978, and the implementation of a two-dose schedule in 2006 led to a substantial reduction in cases [6].

The World Health Organization (WHO) initially aimed to eliminate measles by 2010; however, disparities in vaccination coverage and increasing vaccine hesitancy have hindered progress toward this goal [7]. This article summarizes the global status of measles, epidemiological trends in the United States and Japan, vaccination policies, and differences in immunity assessment methods while discussing future strategies for measles control. Despite substantial progress, measles elimination has still not been achieved owing to uneven vaccination coverage and rising vaccine hesitancy. Herein, we summarize the factors influencing measles resurgence and comment on the effectiveness of current immunization strategies in achieving global measles control.

## 2. Global Status of Measles

Measles is a vaccine-preventable disease; however, it is still a significant global health concern. According to the WHO, the estimated annual number of measles deaths has decreased by 82%, from 772,900 in 2000 to 136,200 in 2022, owing to the introduction and widespread use of measles vaccines [8].

Despite this progress, measles cases have continued to rise in recent years. According to the National Institute of Infectious Diseases, the number of reported measles cases increased worldwide in 2023, an increase of 64% compared with the number of cases reported in 2022, reaching 322,216 reported cases [9]. This surge has been attributed to declining measles vaccination coverage, which dropped to approximately 81% in 2021 owing to delays in immunization programs caused by the COVID-19 pandemic. As a result, the global risk of large-scale measles outbreaks has increased significantly [9].

The highest incidence rates are observed in low- and middle-income countries with low vaccination coverage, particularly in Africa and South Asia, where a substantial proportion of measles-related deaths occur [10]. A 2022 report identified large-scale measles outbreaks in Nigeria, India, Pakistan, and Ethiopia and attributed this resurgence to declining vaccination rates [11]. The COVID-19 pandemic further exacerbated the situation by disrupting routine immunization programs, thereby increasing measles transmission [12].

The WHO defines measles elimination as “the absence of sustained measles virus transmission for more than 12 months in a specific geographic area” [13]. Although the initial goal was to eliminate measles by 2010, this objective has remained unmet in many countries due to regional disparities in vaccination coverage, limited healthcare access, and vaccine hesitancy [7,14]. Even in regions where measles has been declared eliminated, such as Europe and the Americas, sporadic outbreaks continue due to imported cases and declining vaccination rates. For example, the 2018–2019 measles outbreak in Europe led to a surge in cases in Romania, Italy, and France, where decreased vaccine uptake was a key contributing factor [15].

Vaccine hesitancy and misinformation have significantly contributed to measles resurgence. The spread of unscientific information through social networking services and online platforms has led to declining vaccination rates, particularly in high-income countries [16]. In Europe, the European Center for Disease Prevention and Control reported approximately 1500 measles cases in 2024, with unvaccinated individuals accounting for nearly 60% of those cases [17].

Recent data highlight that two-dose measles vaccination coverage remains insufficient worldwide. A global analysis by Plans-Rubió [18] found that in 2023, the average two-dose vaccination coverage was below the 95% threshold needed to interrupt measles transmission in all WHO regions, and the mean prevalence of measles-protected individuals was only 87.6% in the target vaccination population. Furthermore, only the Western Pacific and European regions achieved herd immunity levels sufficient to prevent the transmission of more infectious measles virus variants (R_0_ ≥ 15). Alarmingly, global coverage declined by 3.7% between 2019 and 2023, and the number of zero-dose children remains off track in meeting the Immunization Agenda 2030 (IA2030) goals [18].

Overall, measles remains a global challenge. Sustained vaccination programs, efforts to counter misinformation, and equitable vaccine distribution are critical to achieving long-term measles control and elimination.

## 3. Measles Situation in the United States

Before the introduction of the measles vaccine in 1963, an estimated 3 to 4 million people contracted measles annually in the United States, with approximately 500 deaths each year [19]. Owing to the highly contagious nature of the virus and its cyclic epidemic pattern, outbreaks spread rapidly in communities with susceptible individuals [20].

The introduction of the measles vaccine in 1963 led to a sharp decline in measles incidence and mortality. The average annual number of cases before vaccination (1953–1962) was approximately 530,000. However, by 1966, this number had fallen to 204,136 cases and further declined to 22,231 cases by 1968 [21]. From the 1970s to the 1980s, the annual incidence of measles ranged between 22,000 and 75,000 cases [22].

In the late 1980s, a large measles outbreak occurred in the United States. Between 1989 and 1991, 55,622 cases were reported in total, resulting in 123 deaths [23]. The outbreak was attributed to declining vaccination rates and increased susceptibility among unvaccinated individuals and immunocompromised populations. In response, the Advisory Committee on Immunization Practices (ACIP) revised its measles vaccination guidelines in 1989, introducing a two-dose schedule instead of a single-dose regimen [24].

Following the implementation of the two-dose vaccination program in the 1990s, the number of measles cases declined sharply. In 1994, only 963 cases were reported, decreasing to 301 cases in 1995, 138 cases in 1997, and 100 cases in 1998. Notably, only 29 cases in 1998 were classified as non-imported [25]. In light of this progress, measles was no longer considered an endemic disease in the United States by 1998 [26].

In 2000, the Centers for Disease Control and Prevention (CDC) officially declared measles eliminated in the United States, meaning the country had experienced no sustained measles virus transmission for more than 12 months [13]. However, sporadic outbreaks have continued owing to imported cases.

One of the largest outbreaks in recent history in the United States occurred in 2014, originating at Disneyland in California. The outbreak resulted in 667 reported cases, primarily among unvaccinated individuals or those with unknown vaccination status [27]. In 2019, 1249 cases were reported, marking the highest incidence since 1992, with vaccine hesitancy identified as one of the major contributing factors [28]. In addition, the COVID-19 pandemic significantly disrupted routine healthcare access and immunization programs, contributing to increased measles cases in the United States between 2020 and 2022 [29].

In summary, although the epidemiology of measles in the United States has improved significantly following the introduction of the vaccine in 1963 and adoption of a two-dose policy in 1989, challenges persist owing to imported cases and vaccine hesitancy, leading to periodic outbreaks.

Measles prevention endeavors in the United States began in 1963 with the approval of the measles vaccine and the launch of a nationwide immunization program [24]. In 1967, the CDC initiated the National Measles Eradication Program, marking the start of concerted efforts to eliminate measles in the country [30].

Initially, a single-dose vaccination regimen was recommended. However, owing to inadequate immunity post-vaccination, the ACIP revised its recommendations in 1989, transitioning from a one-dose to a two-dose schedule [24]. The revised schedule stipulated the first dose at 12–15 months of age and the second at 4–6 years of age, improving immunity acquisition from 95% to 99% [31].

By 1981, mandatory vaccination policies led to 97% of the children entering kindergarten and elementary school having received measles vaccinations [32]. By 1998, 55% of U.S. states had enacted laws requiring two doses before school entry, further increasing vaccination coverage [33]. Between 1999 and 2000, the two-dose program significantly reduced the number of measles cases, leading to the official declaration of measles elimination in the United States in 2000 [13].

As of 2010, measles vaccination coverage remained high, with first-dose coverage of 94% and second-dose coverage exceeding 90% [34]. However, vaccine hesitancy, particularly among specific religious communities and individuals opposing vaccination for personal reasons, has contributed to regional disparities in vaccination rates [16].

The 2014 Disneyland outbreak in California underscored these issues, as most cases involved unvaccinated individuals, with low coverage in certain communities facilitating the spread [27]. Similarly, the 2019 outbreak, which resulted in 1249 reported cases, was directly linked to vaccine hesitancy [28].

In response, California enacted a legislation in 2015 that eliminated personal belief exemptions for vaccinations [35]. Following this policy change, vaccination coverage in the state increased from 92% in 2014 to 95% in 2018 [36].

To strengthen immunization efforts, the CDC mandated two doses of the measles vaccine for school entry nationwide [37]. Higher vaccination rates are expected to help mitigate the resurgence of measles cases linked to imported infections.

## 4. Measles Situation in Japan

Historically, measles was a major public health concern in Japan. In the 1950s, several thousand people died from the disease annually [38]. A sentinel surveillance system for measles was introduced in 1981, and no accurate incidence data are available for the time before 1981 [39].

Between 1982 and 1984, the annual incidence of measles ranged from 24.0 to 57.8 cases per sentinel site, with more than 60% of cases occurring among children aged 1–4 years [40]. This trend persisted into the 1990s, with children in this age group still accounting for approximately 60% of the cases reported in 1990 [41]. However, from the late 1990s to the early 2000s, the epidemiological pattern shifted, with an increasing proportion of cases occurring in individuals aged 10 years and older [42]. With the Infectious Diseases Control Law enacted in April 1999, measles became a reportable disease under Japan’s national surveillance system [43], leading to a strengthened surveillance framework and comprehensive data collection.

In 2001, measles cases nationwide were estimated at 286,000, the highest number since 1993 [44]. Among these, 47% occurred among children aged 0–2 years, while 16.6% involved individuals aged 10 years or older [42]. However, between 2003 and 2005, the number of cases declined significantly, reaching 4200 in 2005 [45].

In January 2008, measles was designated a “Nationally Notifiable Disease,” further strengthening reporting requirements [6]. That year, 11,013 cases were reported, but intensified control measures led to a sharp decline, with 732 cases in 2009 and 447 in 2010 [6]. By 2016, the number had dropped to 165, reflecting effective outbreak control [46].

In 2019, Japan recorded 745 cases, the highest number since 2009 [47]. Notably, 70% of cases occurred in adults, with infections decreasing among individuals aged 10–19 years but increasing among those aged 20 and older [47]. The COVID-19 pandemic also affected the incidence of measles. From 2020 to 2022, the number of reported cases remained below 10 annually, with only 6 recorded in 2022 [48].

Overall, measles epidemiology in Japan has evolved significantly. Although large outbreaks occurred in the 1990s and early 2000s, increased vaccination coverage and strengthened surveillance under the Infectious Diseases Control Law have effectively suppressed measles transmission in recent years.

In Japan, the measles vaccination program began in October 1978 when the measles vaccine was designated as a routine immunization [48]. Initially, a single-dose schedule was recommended, but discussions on transitioning to a two-dose schedule emerged in the late 1990s, owing to concerns about immunity acquisition rates and epidemiological trends [48].

An amendment to the Immunization Act in 2006 led to the introduction of a two-dose measles–rubella (MR) vaccine schedule, with the first dose administered at 12–24 months and the second at 5–6 years (before elementary school entry) [48]. Between 2008 and 2012, supplementary vaccination campaigns targeted first-year middle school and third-year high school students to enhance population immunity [48].

The two-dose system significantly reduced the incidence of measles. Between 2010 and 2012, vaccination coverage remained high, with first-dose coverage exceeding 95% and second-dose coverage ranging from 92% to 94% [49]. By 2015, the coverage rates reached 96.2% for the first dose and 92.9% for the second [50]. In 2018, the coverage rates peaked at 98.5% for the first dose and 94.6% for the second [51].

Despite these achievements, cases resurged in 2019, during which time 745 cases were reported [47]. A substantial proportion occurred among adults, with individuals aged 20 years and older accounting for 70% of the cases [47]. This resurgence was attributed to unclear vaccination histories and waning immunity among adults [52].

The COVID-19 pandemic affected routine immunization, leading to a decline in coverage. In 2021, first-dose coverage dropped to 93.5%, while second-dose coverage fell to 93.8% compared with peak levels in 2018 [48].

A comparison of major milestones in measles vaccination policies between Japan and the United States is presented in Table 1. The United States introduced a two-dose schedule earlier (1989) than Japan (2006) and declared measles elimination in 2000, whereas Japan achieved this in 2015. These differences highlight variations in public health strategies and disease burden between these countries [53].

## 5. Differences in Immunity Assessment Methods for Measles in the United States and Japan

### 5.1. Overview

The assessment of measles immunity differs between the United States and Japan in several aspects. Immunity is generally evaluated by measuring the presence and titer of measles-specific IgG antibodies in serum. However, the target populations, assessment methods, and verification of vaccination history differ.

### 5.2. Immunity Assessment Methods in the United States

In the United States, the CDC provides guidelines for assessing measles immunity. According to the 1989 recommendations by the ACIP, individuals are considered immune if they meet one of the following criteria [54]:

- Born before 1957, as they are likely to have acquired natural immunity through infection;
- History of physician-diagnosed measles;
- Presence of measles-specific IgG antibodies confirmed by serological testing;
- Documented receipt of one or two doses of the live measles vaccine.

Serological testing for measles antibodies is recommended for healthcare workers and individuals without documented vaccination. If vaccination history is unknown, one or two additional doses of the measles vaccine are advised [24].

In the United States, an enzyme immunoassay (EIA) is widely used for detecting measles-specific IgG antibodies in clinical and public health settings [55]. EIA is used as the standard serological test owing to its simplicity, cost-effectiveness, and broad availability. The plaque reduction neutralization test, once considered the gold standard for precise immunity assessment, is now rarely used in routine clinical testing. It is primarily utilized for research or outbreak investigations requiring highly specific immune assessment [56].

### 5.3. Immunity Assessment Methods in Japan

In Japan, measles immunity is also evaluated by measuring serum antibody titers. However, unlike in the United States, serological testing is more commonly used for routine immunity assessment rather than primarily relying on vaccination history [57].

The three main serological testing methods used in Japan are as follows:

- EIA: Used for general screening, similar to its application in the United States, but has limitations in sensitivity and specificity;
- Hemagglutination inhibition (HI) test: A cost-effective and simple method suitable for large-scale population surveys, though it has lower specificity;
- Neutralization test (NT): The most accurate method, with high sensitivity and specificity, but is time-consuming and labor-intensive.

Although healthcare workers and adults with an unknown vaccination history may be advised to undergo antibody testing, immunity status in Japan is often evaluated based on antibody titers rather than vaccination records [58]. HI testing is frequently used for large-scale immunity surveillance, whereas EIA and NT are more commonly applied in clinical and detailed immunity assessments [59].

In the United States, immunity assessment is primarily based on vaccination history, and serological testing is only recommended in specific cases (e.g., healthcare workers, individuals with uncertain vaccination history, or during outbreaks). In contrast, Japan relies more on antibody titer measurement, particularly for adults with unclear vaccination histories [59].

## 6. Impact of COVID-19 on Measles Epidemiology and Vaccination Coverage

The COVID-19 pandemic significantly affected measles epidemiology and vaccination coverage. Although international travel restrictions and the reallocation of healthcare resources led to a temporary decline in reported measles cases, this was likely owing to reduced surveillance and diagnostic activities rather than an actual decrease in the prevalence of infections. In addition, disruptions in routine immunization programs have widened immunity gaps, increasing the risk of future outbreaks [11,60,61].

During the COVID-19 pandemic, global travel restrictions reduced the number of imported measles cases, leading to a temporary decline in reported cases in some countries. However, this did not indicate measles elimination; rather, the decline likely resulted from delays in diagnosis and weakened surveillance systems [62].

- Global situation: Between 2020 and 2021, the number of reported measles cases decreased, but since 2022, case numbers have been rising owing to an increase in the population of unvaccinated individuals [13]. The WHO has warned that the decline in measles vaccination coverage in 2021 could elevate the risk of global outbreaks in the coming years [63].
- United States: From 2020 to 2021, the number of imported measles cases decreased, and domestic case numbers remained low [64]. However, since 2022, declining vaccination coverage has led to an increase in imported cases, with small-scale outbreaks reported in certain regions [65].
- Japan: Between 2020 and 2022, the number of annually reported measles cases remained below ten [48], largely owing to international travel restrictions. However, since 2023, concerns have arisen regarding a potential increase in imported cases [48].

The COVID-19 pandemic led to a global decline in measles vaccination coverage [66]. Factors contributing to this decline included reduced healthcare visits, disruptions in immunization programs, and increased vaccine hesitancy among parents.

Changes in vaccination coverage in the United States:

- Between 2020 and 2021, routine childhood immunization rates declined, with first-dose coverage falling below 90% in some states [66].
- In 2022, national first-dose coverage in the United States was 92.6%, while second-dose coverage was 89.9%, falling short of the 95% threshold required to maintain measles elimination [67].
- The increasing number of unvaccinated children has led to heightened concerns about a greater risk of future outbreaks [68].
- Changes in vaccination coverage in Japan:
- Between 2020 and 2021, MR vaccine coverage in Japan declined, with first-dose coverage at 93.5% and second-dose coverage at 93.8%, both below the 95% target [48].
- Although some recovery was observed in 2023, the growing number of unvaccinated adults remains a public health concern [48].

## 7. Diagnosis

The diagnosis of measles is based on a combination of clinical symptoms, serological testing, and molecular diagnostic methods. Clinically, patients with measles presenting with fever, cough, conjunctivitis, and rhinorrhea, followed by the characteristic appearance of Koplik spots on the oral mucosa, are considered pathognomonic for measles [48]. A generalized maculopapular rash typically develops within 3–5 days of symptom onset, beginning on the face and progressing to the trunk and extremities [63].

Serological testing is commonly used for laboratory confirmation of measles-specific IgM and IgG antibodies. The presence of IgM antibodies suggests recent infection and a fourfold rise in IgG antibody titers between acute and convalescent sera confirms the measles diagnosis [64].

Molecular diagnostic methods, particularly reverse transcription polymerase chain reaction, have high sensitivity and specificity in detecting measles virus RNA. This test can be performed using various samples, including blood, nasopharyngeal swabs, throat swabs, and urine [65]. In addition, virus isolation and genotyping are crucial for tracing infection sources, monitoring transmission patterns, and identifying imported cases [48]. Genetic analysis plays a key role in measles outbreak investigations and public health responses [66].

## 8. Discussion

Measles remains a vaccine-preventable disease and significant global public health challenge. Although outbreaks persist in low- and middle-income countries owing to insufficient vaccination coverage, high-income countries face an increasing risk of measles resurgence owing to vaccine hesitancy and declining immunization rates [7,8].

We compared the epidemiological changes, vaccination policies, and differences in immunity assessment methods for measles in the United States and Japan, as well as examined the impact of the COVID-19 pandemic on measles outbreaks and vaccination rates.

In the United States, the introduction of the measles vaccine in 1963, followed by the implementation of a two-dose vaccination schedule in 1989, significantly reduced the incidence of measles, leading to the official declaration of measles elimination in 2000 [13,24]. However, vaccine hesitancy and imported cases contributed to large outbreaks in 2014 and 2019 [27,28]. In Japan, despite the introduction of the measles vaccine in 1978, outbreaks continued until the adoption of a two-dose vaccination schedule in 2006 and implementation of a nationwide case-reporting system in 2008, which helped control measles transmission [46]. However, while Japan declared measles elimination in 2015, challenges persist, particularly owing to unclear vaccination histories among adults [47]. Ensuring accurate vaccination records remains a priority.

In Japan, measles immunity is primarily assessed using EIA, i.e., by measuring serum antibody titers. In contrast, in the United States, immunity assessment is generally based on documented vaccination history, with serological testing recommended only in specific cases, such as for healthcare workers or individuals with unknown vaccination status [57,58,59]. These methodological differences may contribute to inconsistencies in immunity evaluation and surveillance accuracy. Given the increase in international travel, global cooperation is essential for maintaining measles elimination.

The COVID-19 pandemic significantly affected measles epidemiology and vaccination rates. During the pandemic, international travel restrictions led to a reduction in the number of imported cases, leading to a temporary decline in reported measles cases in some countries [11,61]. However, this decline was likely due to reduced surveillance and diagnostic activities, and since 2022, the risk of measles outbreaks has increased [13,62]. In addition, the decline in vaccination coverage has become a critical concern. In the United States, second-dose measles vaccination rates dropped to 89.9%, and in Japan, MR vaccine coverage remained below the 95% target [48,68].

To prevent outbreaks in the future, maintaining vaccination rates at or above the 95% threshold recommended by the WHO is crucial as this aligns with the estimated herd immunity threshold (92–95%) required to interrupt measles virus transmission [8]. Mathematical modeling suggests that achieving at least 95% immunity by the age of five is necessary for sustaining measles elimination, particularly among school-age children (5–9 years), who play a key role in virus transmission [8]. Epidemiological studies from the 1989–1991 U.S. measles resurgence indicated that vaccine coverage of approximately 80% by the second birthday, combined with 93% population immunity among those aged 6 years and older, could prevent widespread transmission [69].

Recent global analyses further emphasize the widening gap between current vaccination coverage and the levels required for herd immunity. A 2025 study by Plans-Rubió reported that in 2023, the global average for two-dose measles vaccination coverage was only 65.3%. Only the Western Pacific and European WHO regions reached levels sufficient for blocking the transmission of highly infectious measles virus strains (R_0_ ≥ 15). The mean prevalence of measles-protected individuals in the target population was 87.6%, falling short of the 95% benchmark. Furthermore, between 2019 and 2023, coverage indicators declined: the proportion of countries achieving ≥95% two-dose coverage dropped by 39.6%, and the number of zero-dose children remained off track for the AI2030 goal [18].

Measles has one of the highest basic reproduction numbers (R_0_) among infectious diseases, ranging from 12 to 18 [69]. This high transmissibility underscores the need for maintaining high population immunity across all age groups, particularly among adolescents and young adults, who have been increasingly affected by recent outbreaks.

Given the risk of imported cases and declining vaccination coverage, continued efforts to promote vaccination, enhance immunity surveillance, and reinforce public health messaging are imperative. Standardized immunity assessment methods and strong international collaboration are essential for achieving and sustaining measles elimination worldwide.

A recent report of data from the WHO European Region highlighted a sharp resurgence of measles, with over 60,000 cases and 13 associated deaths reported in 2023, predominantly in Azerbaijan, Kazakhstan, Kyrgyzstan, Romania, the Russian Federation, and Türkiye [70]. The report identifies persistent immunity gaps, suboptimal vaccination coverage, and delayed outbreak response as key drivers of these outbreaks. To address this challenge, the WHO has underscored the urgent need for renewed political commitment, comprehensive surveillance systems, and intensified routine and catch-up immunization efforts, especially in areas with historically low coverage. These findings emphasize that even regions previously close to elimination remain vulnerable to large-scale outbreaks unless high population immunity is maintained. Strengthening regional and global coordination as well as tailoring vaccination strategies to local epidemiological contexts will be critical for moving toward sustainable measles elimination.

In conclusion, although this review did not employ a formal comparative study design, it provides a descriptive analysis of measles epidemiology, vaccination policies, and immunity assessment methods in the United States and Japan. These insights may help us identify key factors contributing to differences in measles control and underscore the importance of sustained immunization efforts and international cooperation to achieve and maintain measles elimination.

## Figures and Tables

**Table 1:** Timeline of major measles vaccination policy milestones in Japan and the United States.

Year	Japan	USA
1963		Vaccine Approval
1967		Routine One-Dose Start
1978	Vaccine Approval and One-Dose Start	
1989		Two-Dose Program Start
2000		Measles Elimination Declared
2006	Two-Dose Program Start	
2015	Measles Elimination Declared	

## Data Availability

Not applicable.
